# Distributed smoothed tree kernel for protein-protein interaction extraction from the biomedical literature

**DOI:** 10.1371/journal.pone.0187379

**Published:** 2017-11-03

**Authors:** Gurusamy Murugesan, Sabenabanu Abdulkadhar, Jeyakumar Natarajan

**Affiliations:** Data Mining and Text Mining Laboratory, Department of Bioinformatics, Bharathiar University, Coimbatore, Tamilnadu, India; Harbin Institute of Technology Shenzhen Graduate School, CHINA

## Abstract

Automatic extraction of protein-protein interaction (PPI) pairs from biomedical literature is a widely examined task in biological information extraction. Currently, many kernel based approaches such as linear kernel, tree kernel, graph kernel and combination of multiple kernels has achieved promising results in PPI task. However, most of these kernel methods fail to capture the semantic relation information between two entities. In this paper, we present a special type of tree kernel for PPI extraction which exploits both syntactic (structural) and semantic vectors information known as Distributed Smoothed Tree kernel (DSTK). DSTK comprises of distributed trees with syntactic information along with distributional semantic vectors representing semantic information of the sentences or phrases. To generate robust machine learning model composition of feature based kernel and DSTK were combined using ensemble support vector machine (SVM). Five different corpora (AIMed, BioInfer, HPRD50, IEPA, and LLL) were used for evaluating the performance of our system. Experimental results show that our system achieves better f-score with five different corpora compared to other state-of-the-art systems.

## Introduction

Automatic extraction of Protein-Protein Interaction (PPI) pairs from literature is an important research area in biomedical information extraction, since PPI plays vital roles in many biological pipelines and processes such as drug discovery, cell proliferation etc.[1]. The earlier approaches for PPI extraction from literature includes pattern matching based techniques [2–4], co-occurrence approaches [5] and machine learning based methods [6–9]. Pattern matching techniques [2–4, 10] utilizes a set of handcrafted rules as patterns to extract the PPI pairs from the corpus. On the other hand, in co-occurrence based methods [5] the protein pairs are extracted from the training corpus as co-occurred genes to find the PPI sentences.

Later, for improving the PPI task with better performance, machine learning based (ML) models [6, 11–15] were introduced. In former ML models, linear features [16–18] were often used for PPI extraction. The commonly used linear features include linguistic features such as lexical, word context, and word distance features. For example, Landeghem et al. [16] proposed a rich-set of features in combination with automated feature selection method for PPI extraction. Liu et al.[18] examined the combination of lexical, syntactic and dependency information based features for PPI extraction. However, the main disadvantage of the above feature-based approaches is that they cannot utilize the structural similarity information in a sentence.

In next stage, various kernel-based methods were used to overcome this problem. These methods use kernel function to represent diverse features in a high dimensional space and calculate the similarity between two entities [19–21]. Among the kernel based methods, tree kernel have the ability to use the structural information from sentences and are mostly used in PPI extraction task [19].Various tree kernels used for PPI extraction task includes sub-tree kernel [21], subset tree kernel [20], partial tree kernel [22], feature-enriched tree kernel [23], etc. Few other kernels used for PPI extraction includes all path-graph kernel [24], and convolution tree kernel [25].

As a recent enhancement, several studies attempt to use multiple kernels to overcome the inadequacy of single kernel. For example, Kim et al.[26] used four kernels namely predicate kernel, walk kernel, dependency kernel and hybrid kernel for PPI prediction based on the sentential structures in two entities. Miwa et al. [27] used lexical features and several parsers combined using composite kernel, which in turn combines multiple kernels such as bag-of-words (BOW), subset tree and graph kernel. Giuliano et al. [28] proposed the Shallow Linguistic (SL) kernel which combines both local and global context kernel. Yang et al.[29] combined multiple kernels: feature-based kernel, tree kernel, APG kernel and part-of-speech path kernel. Similarly, Li et al.[30]combined three kernels namely, feature-based kernel, tree kernel and semantic kernel to extract PPIs. Chiang et al.[13] applied semantic similarity based features along with random forest classifier for PPI extraction from biomedical literature. Niu et al. [14] used a word similarity model approach in which they created a hybrid model based on relational similarity approach. Chang et al.[8] proposed an interaction pattern generation approach using convolution tree kernel for PPI extraction.

However, in all the above approaches the semantic relation between entities is ignored except Li et al. approach [30]. They used semantic kernel that calculates the protein-protein pair similarity and the context similarity features utilizing two external semantic resources: WordNet and Medical Subject Heading (MeSH).

On the other side, Distributed Smoothed Tree Kernel (DSTK) models are proposed recently that takes the advantage of combining Compositional Distributional Semantic Models (CDSM) with tree kernels [31]. DSTK comprises the distributed trees with syntactic information along with distributional semantic vectors representing semantic information of the sentences or phrases [31]. DSTK transfers the sentences into matrices that can then be used by learning algorithm as features. The DSTK model was successfully demonstrated to text classification problem [31].

In this paper, we employ DSTK to extract PPIs from biomedical literature to take advantage of both syntactic structure information and semantic vector representation. Further, to overcome the shortcoming of information loss from single kernel approaches and to utilize the advantage of multiple kernel approaches, the baseline feature based kernel, which uses lexical features such as word features and word distance features is combined with DSTK as a multiple kernel. Both feature base kernel and DSTK are combined using Ensemble SVM for training and testing. Experimental results on five public PPI corpora show that our approach can achieve enhanced performance than other state-of-the art systems.

## Materials and methods

**Fig 1:**
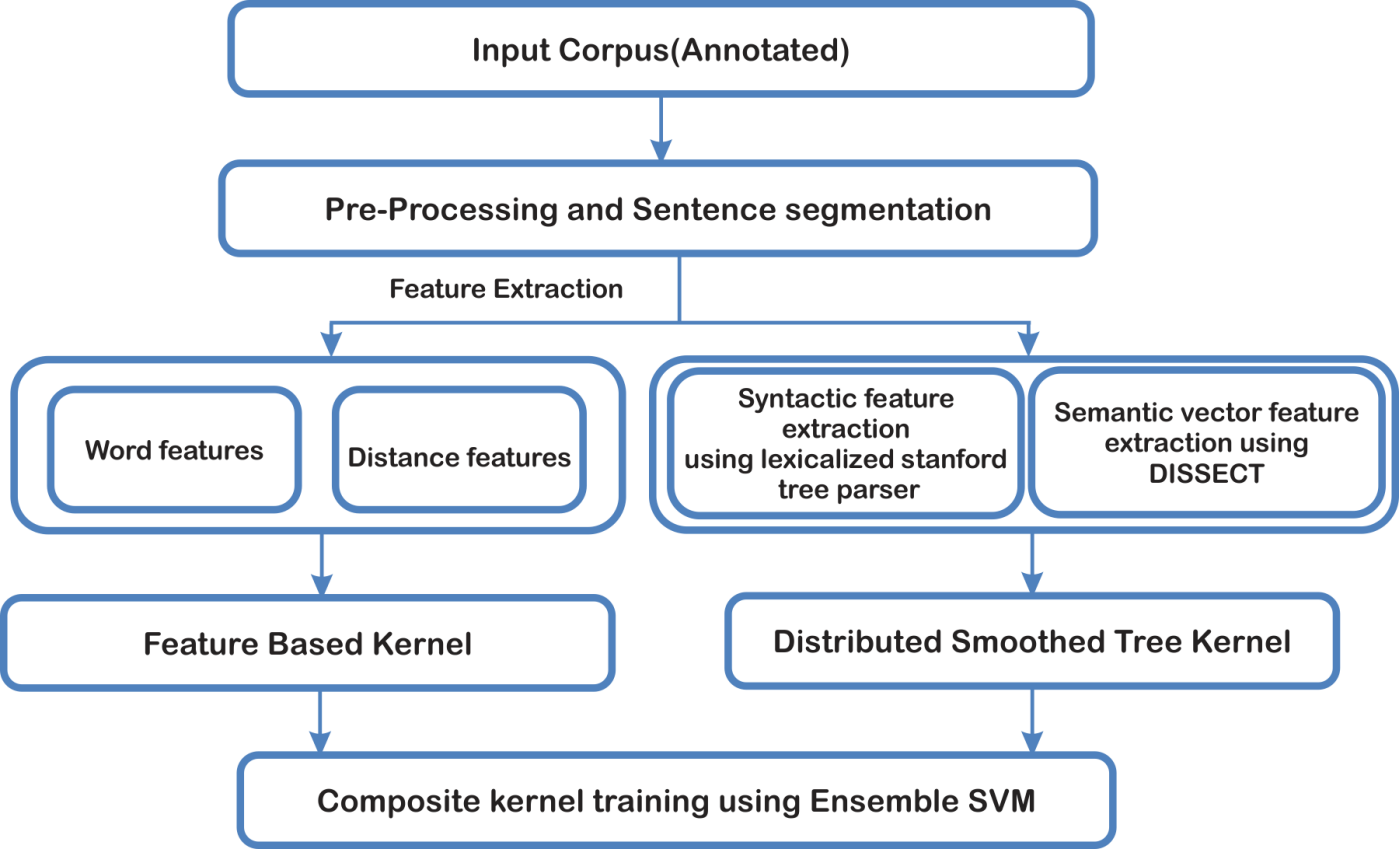
System overview.

Our approach in extracting PPI information comprise of three processing phases. i) Text pre-processing which includes sentence segmentation and data cleaning, ii) Two distinctive types of kernels which includes feature based kernel and distributed smoothed tree kernel iii) Ensemble kernels based learning using SVM. The overall methodology of our approach is shown in (Fig 1) and each component is described in the following sub-sections.

### Text preprocessing

Text preprocessing includes tokenization, sentence segmentation, POS tagging and lemmatization. In addition all the words in the corpus were converted to lowercases and numbers which were found as individual words are replaced with NUM keyword. We used open source tool, OpenNLP [32] for text pre-processing.

### Feature extraction

Different types of features reveal different information aspects of the sentence which contains the PPI instance. In our feature extraction phase we used two distinctive types of kernels i) feature based kernel which uses word context information features and ii) distributed semantic tree kernel which uses distributed trees with syntactic information along with distributional semantic vectors representing semantic information of the sentences or phrases.

#### Feature based kernel

The feature based kernel uses word features and word distance features and is similar to the one previously used for PPI extraction [7,30].

Following are the word features used in our feature based kernel.

*Protein name words*: All the words in the protein names were used as word features.

*Interaction keywords*: The interaction keyword (e.g. bind, regulate etc.) which represent the relation with the protein were used as word feature. If more than one interaction keyword is present, first one will considered as feature.

*Words between two protein names*: All words that are located between two protein names in the interaction sentences were used as word features.

*Surrounding words*: *All* words surrounding the protein names within the word length of 3 were used as word feature.

Similarly, following are the word distance features used in our feature based kernel.

*Number of non-proteins*: The word count of the non-protein words between two protein pairs is considered as one of the count features. If word count is less than 3 the value will be “1”. If word counts between 3 and 6 then the feature value is set to “2”. If the count is 6 to 9 then the feature value is set to “3” otherwise the value is set to “4”. If no words are present in between two protein pairs the value is “0”.

*Number of proteins*: If any protein appears between the two protein pairs in the interaction sentences, the feature value will be set to the count of proteins; if not, the feature value will be set to “0”.

For example, the word and distance feature vector for a sentence “*Biochemical complementation experiments also indicate that the PRP9 and PRP11 proteins interact*.*”* is shown in Table 1.

**Table 1:** Word and distance feature vector.

Feature Names	Feature Values
*Protein name*	P- *PRP9*, p- *PRP11 proteins*
*Words between protein names*	b-and
Words surrounding protein names *Left n words* *Right n words*	l-indicate, l-that, l-the r-proteins, r-interact, r-.
*Interaction keywords*	ik-interact
*No*. *of non-proteins between two proteins*	No. of non-proteins *=* 1
*No*. *of proteins between two proteins*	No. of proteins *=* 0

#### Distributed smoothed tree kernel

We use a special type of tree kernel for relation extraction which exploits both syntactic (structural) and semantic vector information. We adopted Distributed Smoothed Tree kernel (DSTK) introduced by Ferrone and Zanzotto [31]. DSTK merges the distributed trees [33] representing syntactic information with distributional semantic vectors representing semantic information, as used in the smoothed tree kernels [34].

**Fig 2:**
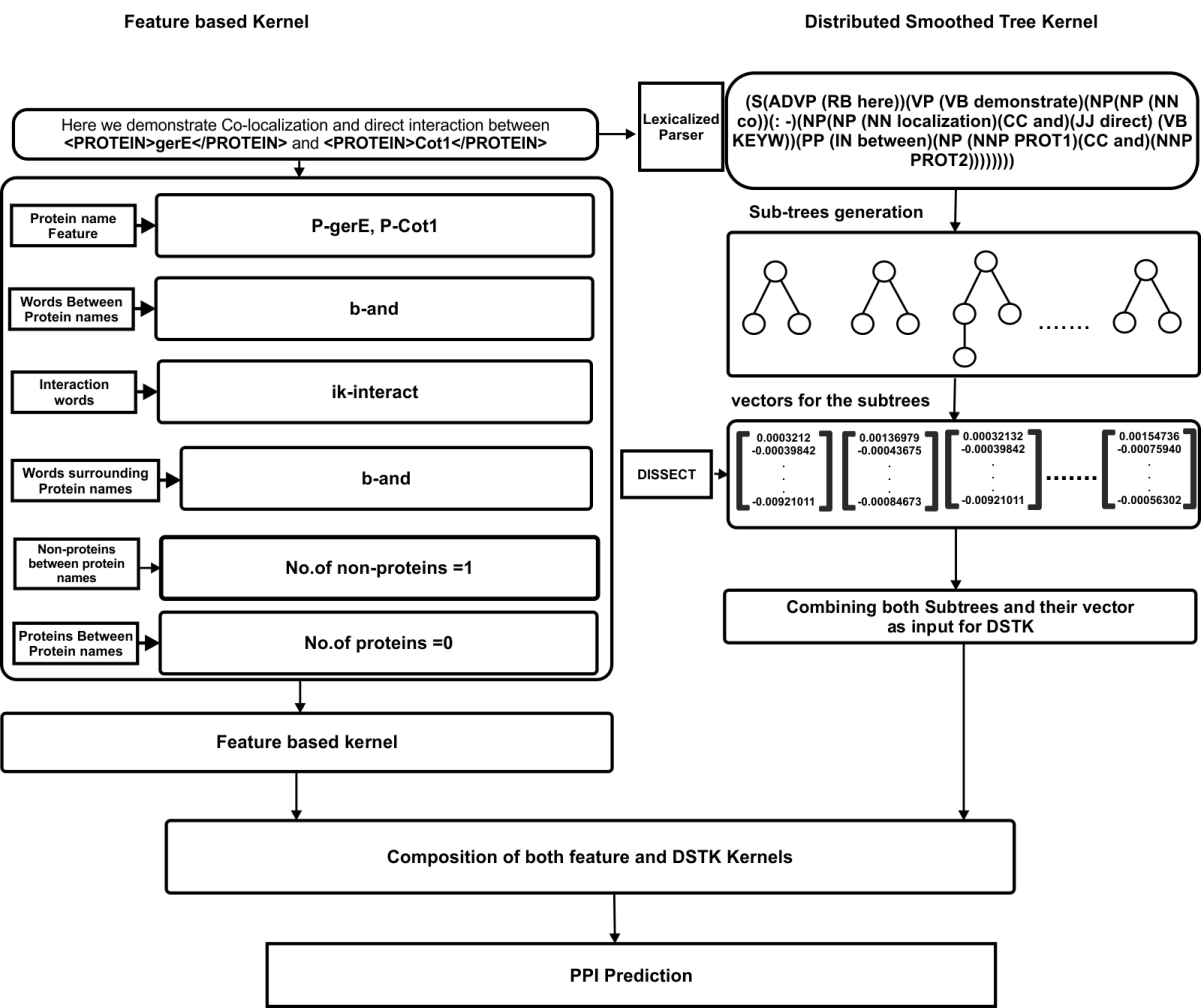
Work flow for feature extraction in both feature based kernel and DSTK.

Hence, DSTK can be considered as a Compositional Distributional Semantic Model (CDSM) and that transforms the sentences into matrices (one dimension encodes the structure and one dimension encodes the meaning) that can be used by the learning algorithm as feature vectors (Fig 2). DSTK is briefly introduced below and for the complete overview refer Ferrone and Zanzotto [31].

DSTK transforms the sentences into matrices that are used by the algorithm as a feature vector. DSTK uses Distributed smoothed trees (DST) to represent the structure and meaning of the sentences.

**Fig 3:**
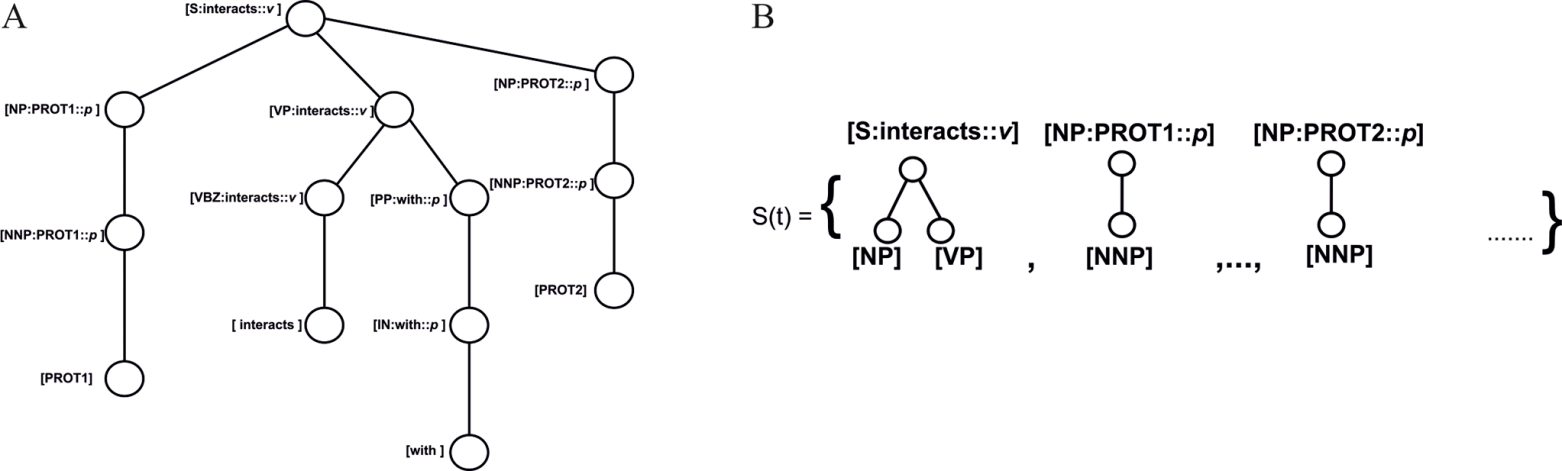
Distributed Smoothed Tree (DST) A) Lexicalized parse tree for DST B) Subtrees of tree in lexicalized parse tree.

DSTs follows the same data structure of constituency-based lexicalized parse trees as shown in (Fig 3A). In (Fig 3A) *N(t)* denotes the set of non-terminal nodes of lexicalized tree *t*. Each non-terminal node *N(t)* has a label *l*_*n*_ composed of two parts *l*_*n*_ = (s_*n*_,*w*_*n*_).

Where s_*n*_ is the syntactic label, while *w*_*n*_ is the semantic headword of the tree headed by n, along with its part-of-speech tag.

DSTs incorporate structure and distributional meaning on a 2D array (a matrix): one dimension determines the structure and one dimension determines the meaning. The structure of a DST is represented as follows: Given a tree *t*, *head(t)* is its root node and *synt*(*t*) is the tree formed from *t* but considering only the syntactic structure (that is, only the *s*_*n*_ part of the labels), *child*_*i*_*(n)* denotes *i*^th^ child of a node n. The distributional vector for the semantic headword *w*_*n*_ is denoted as $\vec{w_{n}}\in R^{k}$.

The lexicalized tree structure is broken into subtrees *t*_*i*_ which is given in (Fig 3B) and belongs to the set *S(t)*. In the set *S(t)*, *t*_*i*_ is a subtree if *synt*(*t*_*i*_) is the subtree of *synt*(t) and *t*_*i*_ contains a node n such that all the siblings of n in *t* are in *t*_*i*_. For each node of *t*_*i*_ DST consider its syntactic label s_*n*_ except for the *head(t)* for which its semantic component *w*_*n*_ is considered. DST can be constructed using the following equation.

$$DST\;(t)=T=\sum_{t_{i}\in S(t)}T_{i}$$ (1)

Where T_i_ is the matrix associated to each subtree *t*_*i*_. The tree (T) can also be defined as follows

$$T=\sum_{t_{i}\in S(t)}T_{i}=\sum_{t_{i}\in S(t)}\vec{synt(t_{i}})\;\vec{head{\left(t_{i}\right)}^{T}}$$ (2)

The similarity between two sub trees using vector representation can be obtained using the Frobenius product between two vector matrices associated with sub trees with the following equation.

$$\begin{array}{c} <T_{i},T_{j}>=<\vec{synt(t_{i}}),\vec{synt(t_{j})}><\vec{head(t_{i}}),\vec{head(t_{j})}>\approx \\ \approx \delta (synt(t_{i}),synt(t_{j}))<\vec{head(t_{i}}),\vec{head(t_{j})}> \end{array}$$ (3)

Using the Eq 3, similarity between lexicalized trees was computed. In order to obtain the Frobenius product, we approximate the dot product between the distributional vectors of headwords defined using the following two scenarios: i) If the subtrees have the same syntactic structure the similarity is obtained as the semantic similarity of their heads ii) If the syntactic structures are different, similarity is defined as 0.

The above scenarios are expressed as

$$<T_{i},T_{j}>\approx \delta \;\left(synt(t_{i}),synt(t_{j})\right).<\vec{head(t_{i}}),\vec{head(t_{j})}>$$ (4)

In order to achieve the above mentioned condition

$$T_{i}=synt\;(\vec{t_{i})}\;\omega \;\vec{{}_{head(t_{i})}}{}^{T}$$ (5)

Where $synt\;(\vec{t_{i})}$ are distributed tree fragment for sub tree *t* and $\omega \;\vec{{}_{head(t_{i})}}$ is the distributional vector of the head. There is a property for distributional tree fragments that

$$\vec{synt(t_{i}}),\vec{synt(t_{j})}\approx \delta (t_{i},t_{j})$$ (6)

Finally by exploiting Eqs (4) and (5) the Eq (4) is satisfied as

$$\begin{array}{c} <T_{i},T_{j}>=<\vec{synt(t_{i}}),\vec{synt(t_{j})}>.<\omega \;\vec{{}_{head(t_{i})}},\omega \;\vec{{}_{head(t_{j})}}>\\ \approx \delta \;\left(synt(t_{i}),synt(t_{j})\right).<\omega \;\vec{{}_{head(t_{i})}},\omega \;\vec{{}_{head(t_{j})}}> \end{array}$$ (7)

Compositional distributional model DST (*t*) computed using the recursive algorithm that utilizes the vectors of the nodes of the tree. It also approximates the smoothed tree kernel and recursively computes the following equation

$$DSTK\;(t^{a},t^{b})=T=\sum_{\begin{array}{c} t_{i}^{}\in S(t^{a})\\ t_{j}^{}\in S(t^{b}) \end{array}}w(t_{i},t_{j})$$ (8)

Where *w*(*t*_*i*_,*t*_*j*_) is the similarity weight between two sub trees *t*_*i*_ and *t*_*j*_. *t*^*a*^,*t*^*b*^ are the lexicalized trees. In DSTK the weights are defined as

$${w\;(t}_{i},t_{j})=\alpha .<\omega \;\vec{{}_{head(t_{i})}},\omega \;\vec{{}_{head(t_{j})}}>.\delta \;\left(synt(t_{i}),synt(t_{j})\right)$$ (9)

Where $\alpha =\sqrt{{⋋}^{\left|N(t_{i})|+|N(t_{i})\right|}}$ and ⋋ is the weight parameter.

Our DSTK implementation for PPI extraction has two parts i) Syntactic tree generation ii) Semantic feature vector generation and discussed below.

*Syntactic tree generation*: To generate syntax tree, we parsed the sentence with Stanford lexicalized Parser [35] and extracted the head words for use in the lexicalized trees with Collins rules [36]. Here we have used interaction keywords as the “head words” or “root”, and constructed the lexicalized dependency tree. Further, in our syntax tree generation, the protein pairs in the sentence were replaced by PROT1 and PROT2 and other protein names occur in the same sentence were replaced by PROT. An example of lexicalized parse tree is shown in (Fig 3A).

*Semantic feature vector generation*: The following steps were used to create the distributional semantic vectors for the sentences in our PPI task.

*Extraction of co-occurrence proteins and their counts*: In this step we used the input corpus to extract co-occurring proteins and their counts along with contextual features such as interaction words and conjunctive features. In our case, any two proteins that are present in the sentences are taken as co-occurred proteins.

*Choosing raw counts that give more similar meaning*: This step involves the application of weighting scheme. The focus here is to probably take out the preferences that commonly influence the counts and to create vectors which better infer similarity in meaning. Pointwise Mutual Information (PMI) is utilized for weighting scheme and Singular Value Decomposition (SVD) is applied to reduce the vector dimension and to eliminate the vectors which were irrelevant to the co-occurrence counts.

For distributed vector generation, we used the concatenation of five input PPI corpora AIMed, BioInfer, HPRD50, IEPA and LLL as our source corpus with a total of about 1.2 billion tokens to extract co-occurring proteins and their counts. The distributional vectors were generated using DISSECT[37] toolkit with standard parameters 1024 and 2048 as the dimension of the distributed vectors and the weight parameter ⋋ was set to 0.4 which was used as the optimal value for most of the previous applications [33]

### Ensemble kernel based learning using SVM

In feature based classification models, ensemble learning approaches demonstrated best performance in many biological applications such as sequence prediction [38, 39], RNA function prediction [40] and literature mining [15, 30]. Further, in most of such data challenges, SVM showed best performance over other classification algorithms. Hence, we employed SVM to train the classifier [41]. We incorporated a composite kernel (*k*_*ckl*_) by combining both feature based kernel (*k*_*fea*_) and distributed smoothed tree kernel (DSTK) (*K*_*DSTK*_).

The ensemble kernel can be obtained by following equation

$$k_{ckl}=k_{fea}+K_{DSTK}$$ (10)

F_1_ and F_2_ are two feature vectors from feature based kernels and D_1_, D2 are the two sub trees of distributed smoothed trees. V_1_ and V_2_ represent the distributional vectors for the sub trees D_1_ and D_2_.The parameter w is the similarity weight between two sub trees D_1_ and D_2_ using vector T_vec_.

$$T(F_{1},F_{2})=T_{v}(F_{1},F_{2})+w(T_{d}(D_{1},D_{2}).T_{vec}(V_{1},V_{2}))$$ (11)

## Results and discussion

### Datasets

To access the performance of our system, we utilized five annotated and publically available PPI corpora namely AIMed [42], BioInfer [43], HPRD50 [3], IEPA [44], and LLL [45]. All the five corpora had various annotating information’s and grouped into frequent layout to extract the PPI’s. The corpus statistics are given in Table 2.

**Table 2:** List of corpora used for evaluation.

S.NO	CORPUS	COUNT	No. of interaction proteins
1	AIMed	1955 Sentences	1000 positive interaction pairs,4834 negative interaction pairs
2	BioInfer	1100 Sentences	2534 positive interaction pairs, 7132 negative interaction pairs
3	HPRD50	145 Sentences	163 positive interaction pairs, 270 negative interaction pairs
4	IEPA	486 Sentences	335 positive interaction pairs, 482 negative interaction pairs.
5	LLL	77 Sentences	164 positive interaction pairs,166 negative interaction pairs

### Evaluation metrics

To explore the performance of the system we used the three different types of metrics commonly used in information extraction problems. These are Precision (P), Recall(R), and F-score. Precision and recall is measured by four metrics true positive (TP), true negative (TN), False positive (FP) and False negative (FN). The final F-score is calculated by the following procedure.

Precision(P)=TP/(TP+FP) (12)

Recall(R)=TP/(TP+FN) (13)

F−Score(F)=2*P*R/(P+R) (14)

**Fig 4:**
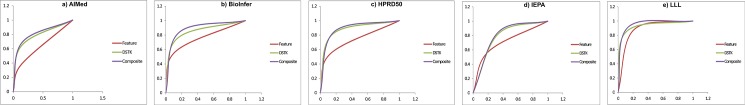
ROC plotting of our three different kernels (feature based, DSTK, Composite) in five corpora a) AIMed b) BioInfer c) HPRD50 d) IEPA e) LLL.

We performed 10-fold cross validation on the datasets to calculate the above metrics. The performance was evaluated by dividing the PPI dataset into ten subsets, for each run, 90% of the data was used as the training set, and the remaining 10% was used as the test set. Then, each of the 10% of the data was selected one by one and tested by the model trained with the remaining 90% of the PPI datasets. The average score was obtained by repeating the above process ten times. In 10-fold cross validation, three types of experiments were performed i) Feature based kernel with linear feature only ii) DSTK with distributed tree features and iii) Composite kernel as combination of both kernels to evaluate the performance of our system. Precision (P), recall (R), F-score (F) results of our three approaches (feature based, DSTK, composite kernel) evaluated by 10-fold cross-validation on five corpora, AIMed, BioInfer, HPRD50, IEPA, LLL is shown in Table 3. It is obvious from the Table 3 that feature based kernel results high precision but low recall. On the other side, distributed models such as DSTK achieves high recall and relatively low precision when compare to simple feature based kernel. The final composite kernel which takes advantage of the both lexical features and distributed tree features improves the overall F-score when compared to single kernel approaches in all five corpora. (Fig 4) shows the ROC plot of three kernels for the entire five corpora.

**Table 3:** Experimental results on three kernel feature based (K_fea_), DSTK (K_DSTK_) and composite (K_ckl_).

Corpus	AIMed			BioInfer			HPRD50			IEPA			LLL		
(%)	P	R	F	P	R	F	P	R	F	P	R	F	P	R	F
K_fea_	73.59	37.43	49.62	79.0	57.12	66.30	68.5	54.5	60.70	80.0	57.23	66.72	87.83	79.14	83.25
K_DSTK_	64.25	68.50	66.30	69.25	**75.15**	72.07	72.30	**80.75**	**76.29**	75.02	**82.71**	**78.67**	**89.64**	85.32	87.42
K_ckl_	**68.91**	**73.24**	**71.01**	**75.7**	**76.90**	**76.29**	76.25	**84.15**	**80.0**	75.85	**85.15**	**80.23**	87.31	**91.18**	**89.20**

### Comparison with other systems

The two main components of our approach include the use of DSTK kernel which utilizes the semantic meaning of the sentence in addition to the syntactic structure and use of multiple kernels. To demonstrate the advantages of our system over other earlier approaches we made comparisons with following three different methods

1. Methods using semantic meaning of sentences
2. Methods using multiple kernels
3. Methods using state of the art non-kernel methods

#### Semantic tree kernel vs. semantic feature kernel

To our knowledge, the only other approach which utilizes semantic meaning of the sentences is work of Li et al.[30]. They used multiple-kernels to extract PPIs, combining three kernels namely feature-based kernel, tree kernel and semantic feature kernel and evaluated on AIMed corpus. Their feature based semantic kernel consists of two features, protein pair similarity and context semantic similarity [30].

Our DSTK kernel based multiple kernel approach outperforms the simple feature based semantic kernel approach and results are shown in Table 4 for AIMed corpus.

**Table 4:** Comparison of our method with (Li et al [30]) in AIMed Corpus.

System	P	R	F
Li *et al* [30](feature + semantic + tree kernels	72.45	66.70	69.46
**Ours** **(feature kernel + DSTK)**	**68.91**	**73.24**	**71.01**

#### Semantic tree kernel vs. other multiple kernels

To demonstrate the advantages of our system over other multiple kernel based methods, we compared our system performance with other state-of-the art systems which uses multiple kernels and also evaluated on all the five corpora. Table 5 show the performance comparison of our method with other multiple kernel based approaches on all five corpora: AIMed, BioInfer, HPRD50, IEPA, and LLL in terms of F-score. The results indicate clearly that our system outperformed all the existing multiple kernel based approaches in all the five corpora which shows the importance of semantic meaning of the sentences in PPI extraction task.

**Table 5:** Comparison of our method with other kernel based methods.

Corpus	AIMed	BioInfer	HPRD50	IEPA	LLL
**Ours** **(feature kernel +DSTK)**	**71.01**	**76.29**	**80.0**	**80.23**	**89.20**
Li et al[15] (feature based+ tree kernel+ features)	69.7	74.0	78.0	76.5	87.3
Miwa et al [27] (BOW+ subtree +graph kernels)	60.8	68.1	70.9	71.7	80.1
Choi et al [46] (convolution parse tree kernel)	67.0	72.6	73.1	73.1	82.1
Satre et al[47] (BOW + shortest path + dependency graph)	64.2	67.6	69.7	74.4	80.5
Satre et al [48] (BOW + dependency graph)	52.0	**-**	**-**	**-**	**-**
Miyao et al[49] *(*BOW + constituent parse tree)	59.5	**-**	**-**	**-**	**-**

#### Semantic tree kernels vs. non-kernel methods

To further demonstrate the advantages of our DSTK system over other non-kernel based methods, we carried out the third evaluation of our method with three recent work on i) deep neural network [9]ii) automatic feature selection [17] iii) deep learning methods [50].

Table 6 shows the comparison results of our method with other non-kernel based methods as mentioned above. To conclude, our approach outperforms all the three non-kernel based approaches on all the five corpora (AIMed, BioInfer, HPRD50, IEPA, and LLL).

**Table 6:** Comparison of our method with other non-kernel methods.

Corpus	AIMed	BioInfer	HPRD50	IEPA	LLL
**Ours** **(feature kernel +DSTK)**	**71.01**	**76.29**	**80.0**	**80.23**	**89.20**
Zhao et al [9] (deep neural network)	56.12	61.26	71.28	74.19	80.99
Phan et al [17] (novel feature selection)	45.1	-	72.6	69.8	76.5
Peng et al[44] (deep learning)	63.5	65.3	-	-	-

In general, while analyzing the results, DSTK performs better than both multiple kernel based approaches and other non-kernel approaches. This may be due to the following facts.

- By refining syntactic tree with semantic vector representation DSTK defines the importance semantics of the sentence in PPI task.
- By implementing lexicalized dependency parsing we generate a verb-centric tree which contains interaction keywords or any other verbs as head words or root. This helps to solve the missing interaction keyword problem while extracting the protein pairs in the tree.
- DST sub tree generation helps in the extraction of multiple interaction pairs in the same sentence.

These facts are further explained in the following example sentence

**Example**:

***Armadillo (Arm) repeat 10** to the COOH terminus of **beta-catenin** is involved in **binding** to **CBP**, whereas **beta-catenin interacts directly with** the **CREB-binding domain of CBP***.

In the above example while extracting protein pairs “*beta-catenin” and “CBP*”, we found feature based kernel tag them as negative because it cannot capture the syntactic representation. However, while applying DSTK, it tags both positive because while extracting protein pairs from the sentences DSTK extracts not only protein pairs but also interaction keyword (e.g. binding) present in the sentence.

Further, the words *“interacts”*, *“binding”* is the head of the two sub trees indicates high possibility of direct interaction. It also helps to extract one-many, many-many, many-one relationship correctly based on the dot product of the sub trees and vector matrix to generate a DST.

In error analysis, we found our system fails to capture PPI information from complex sentences which have four or more protein names. Table 7 shows the examples of such complex sentences in each corpus. Applying a sentence simplification method before parsing such sentences may solve this problem. In the current study, we used Stanford Parser (Klein and Manning 2003) to parse the sentences. However, there are different parsers and each output different syntactic structures. We hope, exploring parser specifically developed and trained in biomedical text will address this issue and also improve system accuracy.

**Table 7:** Complex sentences extracted while annotating PPI.

S.No	Corpus	Interaction sentences
1	BioInfer	Immunopercipitation of metabolically labeled proteins with **<protein>**HECD-1 **</protein>**revealed three bands corresponding to **<protein>**E-cadherin**</protein>**, **<protein>**alpha-catenin**</protein>**, and **<protein>**gamma-catenin**</protein>** and a **<protein>**79-kDa band**</protein>** which was apparently smaller than that of normal **<protein>**beta-catenin**</protein>**, indicating truncated **<protein>**beta-catenin**</protein>**.
2	HPRD	In addition, coexpression of **<protein>** SRC-1**</protein>** but not**<protein>** p300**</protein>** further stimulated the **<protein>**Bcl3**</protein>** -mediated enhancement of the **<protein>** 9-cis-RA**</protein>**-induced transactivations of**<protein>** RXR**</protein>**
3	IEPA	The hydrophilic form of MDP released from the cells on stimulation with **<protein>insulin</protein>** was recognized by antibodies against the inositol 1,2-cyclic monophosphate cross-reacting determinant, indicating that it had been generated by cleavage of its GPI anchor through the action of a **<protein>phospholipase C</protein>.**

## Conclusion and future work

In this paper we elucidated a multiple kernel based machine learning approach using feature based kernel and DSTK to extract the PPI from biomedical literature. We are the first one to explore a kernel which uses the distributional semantics of the sentence in addition to syntactic structure. Experimental results on all five standard PPI corpora shows that our method improve recall substantially and thus results overall high F-score. The results indicate that the importance of semantics of the sentences in addition to syntactic structure in PPI extraction task.

In future expansion, we plan to apply sentence simplification methods for complex sentences and explore the result using DSTK and other kernels. In addition, we wish to incorporate domain knowledge into PPI extraction. We believe by exploring domain specific methods for parsing, semantic vector generation and feature extraction would be helpful in improving the performance.
